# Supplementary material for: Opportunistic CT for Prediction of Adverse Postoperative Events in Patients with Spinal Metastases
Source: Diagnostics (Basel). 2024 Apr 19;14(8):844. doi: 10.3390/diagnostics14080844 (PMC11049489; doi:10.3390/diagnostics14080844)
Supplement: Supplementary file 1 [file diagnostics-14-00844-s001.zip › diagnostics-2911125-supplementary.pdf]

Supplementary Table S1. Bivariate linear regression for hospitalization, and bivariate logistic regression for postoperative complications within 30 days, and reoperations in spinal metastases undergoing surgery (n=196)

	Length of stay			Postoperative complications (30 days)			Reoperation		
**Variables**	**Coefficient (95% CI)**	**Standard error**	**p-value**	**Odds ratio (95% CI)**	**Standard error**	**p-value**	**Odds ratio (95% CI)**	**Standard error**	**p-value**
Age (years)	-0.02 (-0.10; 0.6)	0.041	0.70	0,99 (0.97; 1.03)	0.013	0.93	0.97 (0.94; 1.01)	0.016	0.06
Body mass index (in kg/m^2)	-0.12 (-0.31; 0.06)	0.093	0.19	0.93 (0.87; 0.99)	0.031	**0.03**	0.99 (0.93; 1.08)	0.038	0.99
Duration primary diagnosis till metastatic operation (days)	-0.01 (-0.01; 0.01)	0.001	0.15	0.99 (0.99; 1.01)	0.001	0.81	0.99 (0.99; 1.01)	0.001	0.20
Albumin (g/dL)	-2.57 (-4.08; -1.06)	0.765	**<0.01**	0.38 (0.22; 0.65)	0.105	**<0.01**	1.58 (0.80; 3.12)	0.548	0.19
Male	0.64 (-1.26; 2.55)	0.965	0.51	0.97 (0.52; 1.81)	0.310	0.93	0.93 (0.42; 2.04)	0.374	0.85
Additional Modified Charlson comorbidity	2.04 (0.13; 3.95)	0.967	**0.04**	1.45 (0.76; 2.78)	0.482	0.26	0.61 (0.28; 1.32)	0.241	0.21
Primary tumor growth									
Slow	0.03 (-2.32; 2.38)	1.193	0.98	0.76 (0.36; 1.73)	0.316	0.55	0.58 (0.21; 1.60)	0.301	0.29
Moderate	0.55 (-1.58; 2.68)	1.082	0.61	1.03 (0.52; 2.06)	0.365	0.92	0.63 (0.26; 1.54)	0.286	0.31
Rapid	*Ref*			*Ref*			*Ref*		
Additional metastases	0.50 (-1.52; 2.52)	1.023	0.63	1.62 (0.81; 3.26)	0.577	0.17	0.52 (0.24; 1.15)	0.210	0.11
Spinal pain	2.47 (-5.26; 0.32)	1.414	0.08	0.89 (0.36; 2.21)	0.413	0.80	0.68 (0.23; 1.97)	0.369	0.47
ASIA impairment scale (preoperative)									
Neurological deficit (A, B, C, or D)	*Ref*			*Ref*			*Ref*		
No neurological deficit (E)	-0.77 (-2.63; 1.09)	0.944	0.48	0.54 (0.29; 0.99)	0.169	**0.049**	0.84 (0.38; 1.82)	0.331	0.65
Metastases region									
Thoracic	*Ref*			*Ref*			*Ref*		
Lumbar	-0.62 (-2.78; 1.55)	1.098	0.57	0.85 (0.42;1.69)	0.099	0.64	0.90 (0.36; 2.42)	0.419	0.82
Cervical	1.22 (-3.97; 1.53)	1.395	0.38	0.13 (0.03; 0.58)	0.298	**0.01**	1.41 (0.50; 4.00)	0.750	0.52
Combined	1.04 (-5.54; 3.45)	2.278	0.65	0.48 (0.10; 2.44)	0.400	0.38	*No Value*		
Previous local radiotherapy	0.75 (-1.21; 2.71)	0.995	0.45	1.54 (0.82; 2.90)	0.497	0.18	1.89 (0.87; 4.14)	0.755	0.11
Previous systemic therapy	-1.27 (-3.12; 0.57)	0.934	0.17	1.75 (0.94; 3.25)	0.554	0.08	1.39 (0.63; 3.04)	0.555	0.42
Pathological fracture	0.77 (-1.07; 2.62)	0.936	0.41	1.34 (0.73; 2.47)	0.417	0.35	1.21 (0.56; 2.63)	0.480	0.63
Number of spine levels undergoing operation									
1	*Ref*			*Ref*			*Ref*		
2	-0.93 (-3.51; 1.65)	0.936	0.48	1.73 (0.73; 4.05)	0.751	0.21	0.90 (0.32; 2.49)	0.468	0.84
3 or more	-3.25 (-5.24; -1.27)	1.006	**<0.01**	1.56 (0.80; 3.06)	0.537	0.19	0.36 (0.14; 0.95)	0.179	**0.04**
Type of surgery									
Vertebrectomy or corpectomy with stabilization	*Ref*			*Ref*			*Ref*		
Decompression and stabilization	-0.39 (-2.47; 1.70)	1.059	0.72	0.55 (0.27; 1.12)	0.199	0.10	0.85 (0.35; 2.03)	0.377	0.71

Decompression	-0.68 (-3.53; 2.18)	1.447	0.64	0.85 (0.34; 2.12)	0.396	0.72	1.36 (0.46; 4.02)	0.752	0.57
Stabilization	-0.46 (-4.22; 3.30)	1.907	0.81	1.34 (0.42; 4.25)	0.789	0.62	0.38 (0.05; 3.20)	0.416	0.38
Surgical approach									
Posterior	*Ref*			*Ref*			*Ref*		
Anterior	-0.60 (-3.48; 2.28)	1.460	0.68	1.08 (0.42; 2.81)	0.526	0.88	1.22 (0.38; 3.90)	0.724	0.74
Combined	7.56 (1.82-13.35)	2.923	**0.01**	3.47 (0.56; 21.40)	3.221	0.18	1.38 (0.15; 12.80)	1.565	0.78
Body composition measurements[a]									
Subcutaneous adipose tissue									
Area (cm^2)	-0.01 (-0.01; 0.01)	0.004	0.99	0.99 (0.99; 1.01)	0.002	0.06	0.99 (0.99; 1.01)	0.002	0.61
Visceral adipose tissue									
Area (cm^2)	0.01 (-0.01; 0.02)	0.005	0.47	0.99 (0.99; 1.01)	0.002	0.13	0.99 (0.99; 1.01)	0.002	0.23
Muscle									
Area (cm^2)	-0.01 (-0.03; 0.03)	0.029	0.85	0.99 (0.98; 0.99)	0.006	**0.03**	0.99 (0.98; 1.01)	0.007	0.36

CI=confidence interval; kg/m^2=kilogram per square meter; g/dL=gram per deciliter; ASIA=American Spinal Injury Association; cm^2=square centimeters; Ref=reference value.
***Bold** p-values are <0.05.*
